# Anti-inflammatory and Antioxidant Effects of Sesame Oil on Atherosclerosis: A Descriptive Literature Review

**DOI:** 10.7759/cureus.1438

**Published:** 2017-07-06

**Authors:** Edmund Hsu, Sam Parthasarathy

**Affiliations:** 1 University of Central Florida College of Medicine; 2 Burnett School of Biomedical Sciences, University of Central Florida College of Medicine

**Keywords:** atherosclerosis, sesame oil, antioxidant, cardiovascular disease, anti-inflammatory

## Abstract

Sesame oil (SO) is a supplement that has been known to have anti-inflammatory and antioxidant properties, which makes it effective for reducing atherosclerosis and the risk of cardiovascular disease. Due to the side effects of statins, the current recommended treatment for atherosclerosis and cardiovascular diseases, the idea of using dietary and nutritional supplementation has been explored. The benefits of a dietary health regime have piqued curiosity because many different cultures have reaped health benefits through the ingredients in their cooking with negligible side effects. The purpose of this literary review is to provide a broad overview of the potential benefits and risks of SO on the development of atherosclerosis and its direction toward human clinical use. Current in vivo and in vitro research has shed light on the effects of SO and its research has shown that SO can decrease low-density lipoprotein (LDL) levels while maintaining high-density lipoprotein (HDL) levels. Current limitations in recent studies include no standardized doses of SO given to subjects and unknown specific mechanisms of the different components of SO. Future studies should explore possible synergistic and adverse effects of SO when combined with current recommended pharmaceutical therapies and other adjunct treatments.

## Introduction and background

Atherosclerosis, the formation of plaques in arteries, has been the topic of extensive research due to its critical role in increasing the risk of cardiovascular diseases such as coronary heart disease (CHD) [1-2]. CHD is one of the leading causes of mortality and morbidity in Americans and causes approximately 610,000 deaths in the United States annually. Due to the high prevalence rate of one in four deaths due to CHD [1], many research studies have explored the mechanism of atherosclerotic plaque formation. Studies have come up with two prominent hallmarks of pathogenesis: the accumulation of cholesterol in the endothelial lining of arteries carried by low-density lipoproteins (LDLs) and chronic inflammation due to a high ratio of pro-oxidants to antioxidants [2-3]. Studies have implied that these hallmarks of atherosclerosis are not independent but are part of the same process where the abnormal deposition of LDL-cholesterol (LDL-C) leads to an inflammatory response resulting in fatty plaques and vascular occlusion [4-5].

Previous literature has established that high serum cholesterol, more specifically elevated LDL levels, is a measure of atherosclerotic risk and, therefore, the reduction of plasma LDL levels should reduce the amount of atherosclerosis and its consequent risk of CHD [4]. LDL and high-density lipoprotein (HDL) are carriers of cholesterol. LDL carries cholesterol to tissues in contrast to HDL, which carries cholesterol to the liver for disposal [6]. An increase of LDL in blood plasma can cause an imbalance in lipoproteins that can lead to build-up of cholesterol, especially in the tunica intima of the large and medium-sized arteries [7]. Cells of the vascular wall secrete oxidative products and can initiate lipid oxidation of the LDL. While the oxidized LDL levels increase and accumulate in the artery, damaged endothelial cells release inflammatory signals to initiate an immune response to fight the cholesterol aggregation [2, 8]. This induces oxidative stress, an imbalance between pro-oxidants and antioxidants, and can cause oxidative damage to surrounding endothelial cells [3, 9]. Macrophages sense the signals and infiltrate the tunica intima to phagocytose the oxidized LDL aggregation [10-12]. With a chronic inflammatory response and the accumulation of oxidized LDL, a fatty streak in the arterial tunica intima forms [13]. Over time, the fatty streak grows, thickens the arterial walls, and contributes to chronic inflammation [14]. This leads to the development of a stable plaque, which can be at risk for eruption when it becomes unstable [15].

The guidelines for cholesterol management through the American College of Cardiology/American Heart Association recommend using statin therapy as the primary prevention treatment for managing cholesterol levels [16]. Statins are hydroxy-methylglutaryl coenzyme A (HMG-CoA) reductase inhibitors, which inhibit the HMG-CoA reductase, an essential rate limiting enzyme in the cholesterol synthesis pathway [17]. Previous literature has shown that statins are a powerful treatment option, which at a dose of 80 mg/day can reduce LDL-C by about 47% [18]. The use of statins became more frequent when the Scandinavian Simvastatin Survival Study showed that long-term treatment with simvastatin was safe and improved survival rates in CHD patients [17].

While statin use has continued to grow in popularity, an issue with the statin treatment is the intolerance of the side effects associated with taking the drug [19]. Statin therapy is not for every patient; patients with higher levels of LDL-C are more likely to benefit from statin use than patients with lower levels of LDL-C [20]. About 10% of patients complain about the side effects, such as myalgia, and this decreases their patient compliance [16]. Due to the side effects of statins and other pharmaceutical drugs, the idea of using dietary and nutritional supplementation has gained traction in the scientific community [16]. The benefits of a dietary health regime have piqued curiosity because many different cultures have reaped health benefits through the ingredients in their cooking. For example, the use of sesame oil (SO) in Asian cultures has inspired studies on the dietary benefits of consuming SO. One study found that traditional Korean cooking called bugak prepared with pan-fried unroasted SO has been shown to decrease LDL, triglyceride (TG), and total cholesterol (TC) levels [21]. Experimental studies on rats fed a sesame seed extract exhibited a significant decrease in plasma cholesterol, triglycerides, very low-density lipoprotein (VLDL) cholesterol, and LDL. One study tested the effect of sesamin, a lignan in SO, in humans and found a significant reduction in LDL-C [22]. This study also referenced other literature, stating that sesamin may potentially reduce HMG-CoA reductase activity by altering the amount of cholesterol ester and free cholesterol by decreasing acyl-CoA cholesterol acyltransferase (ACAT) activity [22]. By reducing HMG-CoA reductase activity, sesamin could potentially reduce LDL levels in a similar manner as statin drugs without the myopathy side effects.

While these studies were conducted many years ago, more recent studies into the properties of sesamin have concluded that sesamin and other constituents of SO have antihyperlipidemic effects and can improve many of the biochemical measurements found in a lipid panel. An experiment conducted on rats studied how sesamin influenced lipid metabolism because of the relationship between increased secretions of LDL in rat liver, following a decrease in fatty acid oxidation. This study concluded that sesamin appears to be a potent inducer of hepatic fatty acid oxidation and is an inhibitor of hepatic lipogenic enzyme gene expression by down regulating sterol regulatory element bind protein-1 (SREBP-1), which is a transcriptional factor that regulates gene expression for both fatty acid and cholesterol synthesis [23]. A more recent study extracted SO from Sesamum indicum L. and examined the effects on rabbits fed on a high fat diet (HFD). The study found that the rabbits supplemented with SO had a lower circulating level of LDL [24]. Sesamol is another lignan of SO that has also shown anti-inflammatory and antioxidant properties in studies [25].

All of these previous studies strongly indicate that SO supplementation has anti-atherogenic effects [26]. Despite indications of cholesterol-lowering effects through different constituents of sesame seeds and oil in many animal studies, the potential hypocholesterolemia properties of raw SO have not been tested much, to the best of our knowledge, in human studies.

The purpose of this literary review is to provide a broad overview of the effects of SO on atherosclerosis and its direction toward human clinical use. This review will examine the recent experimental studies that have been specifically conducted on SO and its effects on cholesterol levels and inflammation in animal atherosclerotic models and in vitro interactions between leukocytes involved in atherosclerosis and arterial tissues. The basis for this review is to select scientific research that tests SO or its lignans as a dependent variable in experiments about atherosclerosis and its hallmarks, inflammation or cholesterol. The time frame for research reviewed is from January 2010 to January 2016 in order to distinguish the most recent studies about SO.

Materials and methods

To begin the review article search, search words [SO] and [cholesterol or inflammation or atherosclerosis] were used to begin the search for papers that studied the effects of SO on levels of cholesterol or inflammation or atherosclerosis. The following criteria were used to further narrow down papers published:

1. Published between January 2010 and January 2016.

2. In vivo mammalian animal or human studies or in vitro studies.

3. Tests the use of SO, by itself or combined with other oils.

4. Written in English.

5. Topic pertains to SO’s effects on atherosclerosis, cholesterol build-up, or inflammation.

Results

After the initial search of [SO] and [cholesterol, inflammation, or atherosclerosis] in the PubMed database, a total of 134 papers that included the key terms were found. After narrowing down the results with a time frame from January 2010 to January 2016, the number of studies dwindled down to 43. Afterward, each abstract of the 43 search results were individually scanned with the criteria stated in the methods. The final search yielded 14 studies that experimented on the effects of SO on atherosclerotic risk factors, such as cholesterol levels and inflammation, and key players that initiate or lead to the progression of atherosclerosis. Below is a table of summary of each of the chosen studies (Table 1).

**Table 1:** Table of Results AA: Arachidonic Acid; ABCA1: ATP-binding Cassette A1; ALA: α-linolenic acid; AMBK: Adenosine Monophosphate-activated Protein Kinase; ANCOVA: Analysis of Covariance; ANOVA: Analysis of Variance; CHO: Chinese Hamster Ovary; DMRT: Duncan’s Multiple Range Test; DMSO: Dimethyl Sulfoxide; ELISA: Enzyme-linked Immunosorbent Assay; FMD: Flow-mediated Dilation; GCO: Garden Cress Oil; HDL: High density Lipoprotein; HDL-C: HDL- Cholesterol; HFD: High-fat Diet; hs-CRP: Highly Sensitive C-Reactive Protein; HUVEC: Human Umbilical Vein Endothelial Cell; ICAM-1: Intercellular Adhesion Molecule 1; LA: Linoleic Acid; LDL: Low-density Lipoprotein; LDL-C: LDL-Cholesterol; LDL-C: low-density Lipoprotein Cholesterol; LDLR: LDL receptor; LDLR: Low-density Lipoprotein Receptor; LPS: Lipopolysaccharide; PCR: Polymerase Chain Reaction; PGE2: Prostaglandin E2; RCT: Reverse Cholesterol Transport; SOAE: Sesame Oil Aqueous Extract; TC: Total Cholesterol; TG: Triglyceride; VLDL: Very Low-density Lipoprotein. SPSS Software Package v.10.0. (IBM, NY, USA.)

Study Number	Reference and Purpose of Study	Experimental Design and Methods of Statistical Analyses	Participants, Control Group, and Experimental Group Description	Outcomes Measured [parameters]	Results
1	To evaluate the effect of sesame oil on inflammation, RCT, lipid metabolism, and lesion formation in LDLR knockout mice [27].	1. Feed respective groups of LDLR­^-/-^ knockout mice with respective atherogenic diets for 15 weeks. 2. Sacrifice mice for blood, plasma and tissue samples. 3. Analyze samples by: plasma lipid analysis with Cholestech LDX analyzer, quantification of aortic lesions, cDNA synthesis and PCR, global cytokine and gene array, in vitro oxidation of LDL in presence of sesamol and sesamin. Statistical analysis by: Student *t*-test Prism Pad software’s Wilcoxon matched paired test *P* < .05 = significant	66 four-week-old female LDLR^-/-^ knockout mice that weigh between 18 g and 20 g from Jackson Laboratory. Control: LDLR^-/-^ knockout female mice fed an atherogenic diet that had 17% saturated fat in milk. Experimental: LDLR^-/-^ knockout female mice fed an atherogenic diet that replaced 17% of saturated milk fat with sesame oil.	Percentage change in weight plasma lipid analysis TRG TC LDL VLDL HDL Quantification of aortic lesions by measuring surface area. Gene array in mice livers for lipoprotein signaling and cholesterol metabolism. Gene array in mice aorta for mRNA expression. Cytokines array.	Insignificant weight change Plasma lipid Analysis TRG: C > Exp TC: C > Exp LDL: C > Exp VLDL: C > Exp HDL: C < Exp 30-40% reduction in Exp than C. Quantification of aortic lesions C > Exp in lesion size Gene array in mice livers showed increased expression of genes related to RCT and lipid metabolism in SO fed mice. Gene expression in mice aortas showed that SO fed mice had increased mRNA levels of the RCT gene ABCA1, but reduced levels of ABCG, monocyte markers, and scavenger receptors. Cytokines array showed that sesame oil increased expression of genes pertaining to RCT and cholesterol metabolism.
­2	[28] Selvarajan K, et al. 2015 Apr To evaluate the effect of Sesame Oil Aqueous Extract [SOAE] on inhibiting inflammation and regulating lipid metabolism	1. For LDL oxidation test, oxidizing agents were added to extracted lipoproteins in presence and absence of SOAE. 2. For PCR array, macrophages pretreated with SOAE were incubated with LPS for 24 hours. Analysis by Qiagen array for atherosclerosis. 3. For real-time PCR analysis for inflammatory markers, HUVECS and macrophages were treated with SOAE and LPS/TNF-a. 4. Medium from macrophages resulting from SOAE and LPS treatments was collected and analyzed with ELISA 5. Webster mice were injected with SOAE and LPS intraperitoneally at varying concentrations. 6. After two hours, mice were sacrificed and plasma and tissue were extracted. Plasma and tissues were analyzed. Statistical Analysis by: Student t-test *P* < .05 = significant	8-week Swiss Webster mice, RAW 264.7 cells, human umbilical vein endothelial cells [HUVEC] Control: Cells and mice not treated with sesame oil. Experimental: Cells and mice treated with aqueous component of sesame oil.	PCR array analysis: Genes upregulated or downregulated between LPS+SOAE, LPS, and SOAE Cytokines analysis ELISA for IL-6 and TNF-α Measure of NF-κB transcription and translocation by PCR array immunofluorescence Oxidation of lipoproteins measured in absorbance over time. Transport gene expression analysis by luciferase and GFP activity.	PCR array showed that SOAE reduced LPS-induced inflammation in RAW 264.7 macrophage cells. ELISA showed significantly reduced expressions of IL-6 and TNF-α in macrophages and endothelial cells in a concentration-dependent manner. SOAE was effective in inhibiting LPS-induced TNF-α and IL-6 levels in vivo at different concentrations, transcription and translocation of NF-κB, and oxidation of lipoproteins in vitro. SOAE activates liver X receptors, which regulate scavenger receptors expression and increased ATP-binding cassette A1 [ABCA1] mRNA expression.
3	[29] Majdalawieh AF, et al. 2015 Aug To show the anti-atherogenic effects of: 1. sesamol on Watanabe heritable hyperlipidemic rabbits 2. sesame oil on LDLR^-/-^mice by the sesamol derivative [INV-403] and sesame oil in Watanabe heritable hyperlipidemic rabbits and LDLR-/- mice	1. Co-transfect Chinese hamster ovary cells with respective luciferase constructs with pCMV-B-galactosidase expression vector. 2. After 24 hours of post-transfection, the transfectants were treated with different concentrations of sesamol and sesame oil, with DMSO as a negative control. 3. To test whether MAPK pathway is affected by sesamol and sesame oil, using the same preparation stated in 1, 100 uM sesamol and 10 lg/ml sesame oil were added to their respective macrophages in the presence or absence of 10 uM of a MAPK inhibitor. 4. To test whether macrophage efflux improves with sesamol and sesame oil treatment, macrophages were treated with different varying concentrations of sesamol and sesame oil for 24 hours. Then they were induced to uptake cholesterol. Amount of cholesterol efflux was recorded 5. To test whether MAPK signaling pathway is involved in the effects of sesamol and sesame oil on PPARg1 and LXRa transcriptional activity, macrophages were treated with DMSO, sesamol, or sesame oil and MAPk inhibitor. Then they were induced to uptake cholesterol. Amount of cholesterol efflux was recorded Statistical Analysis Student unpaired T-test. P<0.05, P<0.01, and P<0.001 are significant.	[1] Watanabe heritable hyperlipidemic rabbits [2] LDLR^-/-^ mice [3] C57/BL6 mice, chow fed for 8-12 weeks on chow-diet, injected with 4% thioglycolate broth solution intraperitoneally. Killed five days later to collect peritoneal macrophages [4] Chinese hamster ovary [CHO] cells, transfected at 60-80% confluency for luciferase reporter assays and plasmid DNA Control: Co-transfected peritoneal macrophages treated with DMSO Experimental: Co-transfected peritoneal macrophages treated with sesamol or sesame oil	Tests effects of sesamol and sesame oil on regulation of transcriptional activity and expression PPARg1 and LXRa genes. Measured by transcriptional activity and expression in luciverase reporter and B-galactosidase assays Tests effects of sesamol and sesame oil on cholesterol efflux Measured cholesterol efflux by H-cholesterol efflux assay.	Sesamol and sesame oil up-regulate PPARg1and LXRa expression ~1.7 fold compared to control 100 uM Sesamol and 10ug/ml sesame oil enhance PPARc1 and LXRa transcriptional activity in a MAPK-dependent manner Sesamol and sesame oil enhance PPARc1 and LXRa transcriptional activity in a temporal fashion, and this enhancement is statistically significant at all tested time points except for the 6 h treatment. Sesamol and sesame oil augment macrophage cholesterol efflux in a MAPK-dependent manner
4	[30] Karatzi K, et al. 2013 Apr. To examine the possible effects of sesame oil consumption on endothelial function, postprandial and long term intake.	1. Hypertensive male volunteers must fast 12 hours from foods, cigarettes, and medication, and 24 hours from liquids. Blood was collected and an ultrasound was used to determine endothelial function. Patients were given their oil [control or sesame] and directions on ingesting it. 2. Patients were given bread to eat. Two hours after ingestion, patient blood samples and ultrasound was done again. 3. Patients enrolled in the chronic phase of the study were assigned to ingest sesame oil as salad dressing for 60 days. At systematic time intervals of the study, Statistical Analysis by SPSS 18 for Windows Kolmogorov-Smirnov test and Q-Q plots - Tested all continuous variables for normal distribution ANCOVA Two-samples and paired samples student’s t-tests - differences in measured variables between specific time points were evaluated by grouped comparisons P <0.017 = significant	Hypertensive males between 40-65 yr old. Hypertensive males had mean day blood systolic blood pressure >135mmHg and diastolic blood pressure >85 mmHg] and were receiving antihypertensive medication. Control: Hypertensive men on control oil Experimental: Hypertensive men on sesame oil	Postprandial effects of sesame oil Measured by flow-mediated dilation [FMD] and ICAM Long-term effects of sesame oil on FMD and ICAM-1	Postprandial and long-term effects of sesame oil increased FMD from baseline comparison. ICAM decreased significantly at 60 days in the long term study. ICAM did not decrease significantly for the short term postprandial
5	[31] Abdel-Daim MM, et al. 2016 Jan To evaluate the protective and antioxidant potential of sesame oil [SO] and/ or α-lipoic acid [ALA] against DZN toxicity in male Wistar albino rats	1. Male Wistar rats were allowed to acclimate for two weeks before experimentation 2. Rats were injected with their respective fluids. 3. After one hour, all groups of rats were injected with DZN. 4. After four weeks, the rats were sacrificed and samples were collected from the blood, liver, kidney, and heart. 5. Analysis of samples by lipid profile. Statistical Analysis by GraphPad Prism statistical package version 5.0 for Windows ANOVA *Tukey’s *multiple range tests P ≤ 0.05 = significant	Male Wistar rats, weighing 160-200g, and allowed to acclimate before experiment. Control: Saline or DZN injected rats Experimental: SO, ALA, or SO and ALA injected mice.	Lipid profile compared to DZN TC LDL-C HDL-C TCG	SO and/or ALA supplementation ameliorated the deleterious effects of DZN intoxication.
6	[32] Korou LM, et al. 2014 Mar To determine whether ingesting NAC and sesame oil beneficially induces hypolipidemic and antioxidant effects in a hyperlipidemic murine model	1. Mice were fed one of four diets depending on what experimental group they belonged to. 2. After eight weeks, plasma, blood, aortic and hepatic tissues were collected. 3. To assess data: - a lipid profile for plasma - measurement kits for peroxide and NO amount - hematoxylin-eosin staining of aorta and liver Statistical Analysis by: 2-way ANOVA - Measured body weight, food consumption, serum lipid, total peroxides, NO and hepatic enzymes levels with diet and NAC intake or diet and sesame oil ANCOVA - compare T1 measurements after controlling for baseline levels. 1-way ANOVA - Hypothalamic GR protein levels and plasma corticosterone levels Kruskall-Wallis test Mann-Whitney’s U test - Histological scores Benjamini and Hochber’s False Discovery Rate - Assess differences between multiple groups, as well as to control family-wise error to a , 0.05 P ≤ 0.05 = significant	12-week-old male C57bl/6 mice Control: [NC] – C57bl/6 mice on basal diet Experimental: [HC] - C57bl/6 mice on high cholesterol diet [ 2% cholesterol and 0.5% cholic acid ]for eight weeks [HCN] - C57bl/6 mice on high cholesterol diet with NAC supplementation [230 mg/kg p.o.] [HCS] – HCS C57bl/6 mice fed high cholesterol diet enriched with 10% sesame oil for 8 weeks	Lipid profile TC LDL-C HDL-C TCG Total Serum concentrations of: Peroxides NO Hematoxylin and eosin staining of aortic and hepatic tissues	Higher serum levels of total and LDL-cholesterol were recorded in all groups fed the high cholesterol diet. HCN group showed lower lipid levels compared to HC and HCS groups, where there was no observed difference between HCS and HC groups. HCN and HCS groups had a significant decrease of lipid peroxidation compared to control group, whereas only NAC restored NO bioavailability. Lesions observed in HCN group were less severe than those seen in the other high cholesterol groups
7	[33] Sharma AK, et al. 2012 Nov. To determine the role of sesamol in chronic high-cholesterol/high-fat diet [HFD]–induced CMetS in rats.	1. Rats were fed with HFD [55% calorie from fat and 2% cholesterol] for 60 days. 2. On the 30th day, rats with total cholesterol >150 mg/dl were administered sesamol 2, 4 and 8 mg/kg per day for the next 30 days. Statistical Analysis by SPSS software package version 11.5. 1-way ANOVA Bonferroni post hoc test. P < .05 = significant.	Male Wistar albino rats weighing 140-170g and are 6-8 weeks old. Control: Rats fed with regular chow Rats fed with high fat chow diet Experimental: Rats fed with pioglitazone 10 mg/kg per day, and rats on high fat chow diet and varying amounts of sesamol.	Serum analysis glucose, total cholesterol [TC], triglycerides [TG], low-density lipoprotein cholesterol [LDL-C], high density lipoprotein cholesterol [HDL-C], ELISA insulin, leptin, adiponectin, resistin, IL-6, hs-CRP TNF-α.	Rats on sesamol diet had treatment decreased IR, insulin, glucose, lipids, TNF-α, IL-6, leptin, resistin, highly sensitive C-reactive protein [hs-CRP], hepatic transaminases and alkaline phosphatase. Sesamol fed group also had normalization of adiponectin, nitric oxide and arterial pressures in a dose-dependent fashion. Compared to other rat groups, sesamol fed rats showed normal liver effects, which was very apparent at 8 mg/kg Sesamol increased hepatic PPARγ, PPARα and e-NOS protein expressions and decreased LXRα, SERBP-1c, P-JNK and NF-κB expression.
8	[24] Asgary S, et al. 2013 Sept. To evaluate the effects of sesame seed and sesame oil on serum lipids, apolipoproteins, liver enzymes, glucose, and insulin in a hyperlipidemic rabbit model	1. Allow rabbits to acclimate for two weeks 2. Feed rabbits on their respective diets for eight weeks. 3. Analyze serum cholesterol levels. Statistical Analysis by SPSS software version 13.0 Kruskal-Wallis test. Dunn’s Test P <0.05 = significant	Adult male rabbits of New Zealand strain [1.25-2.50kg] Control: Rabbits on normal diet or hypercholesterolemic diet Experimental: Rabbits on hypercholesterolemic diet with 10% sesame seeds or hypercholesterolemic diet with 5% sesame oil	Serum cholesterol TC HDL-C LDL-C+VLDL-C	Supplementation with sesame oil, but not sesame seed, can ameliorate serum levels of lipids and hepatic enzymes in rabbits under a high-fat diet
9	[34] Reena MB, et al. 2011 Jan. To study the effect of blended and inter esterified oils with similar fatty acid compositions on gene expression involved in cholesterol homeostasis in rat liver.	1. Rats were placed on a high fat diet with respective oil diets with single or blended oils. The diet consists of 10% fat from native oils; coconut oil [CNO], rice bran oil [RBO], or sesame oil [SESO]; blended [B]; CNO+RBO[B] or CNO+SESO[B] and inter esterified oil [I]; CNO+RBO[I] or CNO+SESO[I] for 60 days 2. Transcriptional profiling of genes involved in cholesterol homeostasis was studied after feeding rats with a semi purified diet Statistical analysis by SPSS statistical software 1-way ANOVA followed by Duncan’s multiple range test. Pearson correlation coefficients were calculated. P ≤ 0.05 = significant.	Male Wistar rats weighing 40-45g in groups of six Control: Rats fed on diets with single oils Experimental: Rats fed on diets with blended oils.	Serum cholesterol TC HDL-C LDL-C+VLDL-C Fatty acids measurements Expression of genes	Hepatic LDL receptor [LDLR] expression significantly increased in rats fed inter esterified oils by 100–200% compared with rats fed blended oils and by 400–500% compared with rats fed CNO. Positional alteration in fatty acids of oils used in the diet induced changes in LDLR expression, which was accompanied by parallel changes in cholesterol-7a-hydroxylase [CYP7A1] and SREBP-2 genes
10	[35] Chen WY, et al. 2015 Feb. To study the effects of sesamol on plasma L5 levels and atherosclerosis development in Syrian hamsters and on the L5-induced apoptosis of ECs.	1. To test effects of sesamol on plasma L5, Syrian hamsters were fed respective atherosclerotic diets for 16 weeks. 2. Collected blood, plasma, and tissues 3. Treated endothelial cells with L5 to induce apoptosis 4. treated experimental group with sesamol Statistical Analysis by: Student’s t-test. P < 0.05 = significant	Syrian hamsters, and Human aortic endothelial cells Control: Syrian hamsters that are fed a normal diet. Syrian hamsters fed a high fat diet Human aortic endothelial cells that were not treated with plasma L5 Experimental: Syrian hamster that are fed a high fat diet with sesame oil. Human aortic endothelial cells that were treated with L5	Gel electrophoresis to determine electronegativity of L5 Liquid chromatography of L5 plasma levels Lipid profile Observations of Oil Red O staining to count lesions Fluorescence observation and immunoblot analyses of activated caspase-3 to show apoptosis/ activity levels of LOX-1, MAPK, and eNOS.	Sesamol fed hamsters in showed reduced plasma L5 levels when compared with the HFD group. Sesamol fed hamsters showed smaller atherosclerotic lesion size in the aortic arch compared with the HFD group. In human aortic ECs, 0.3−3 μM of sesamol blocked L5-induced apoptosis in a dose-dependent manner. Cells treated with sesamol showed inhibition of the L5-induced lectin-like oxidized LDL receptor-1 [LOX 1] − dependent phosphorylation of p38 MAPK and activation of caspase-3 and increased phosphorylation of eNOS and Akt.
11	[36] Umesha SS, et al. 2012 Dec. To develop vegetable oil blends with α-linolenic acid [ALA] rich Garden cress oil [GCO] and assess their modulatory effect on lipid metabolism.	1. Wistar rats were fed Native and Garden cress oil combinations at 10% for 60 days diet. 2. Collected blood and tissues 3. Lipid analysis Statistical Analysis by: ANOVA Tukey–Kramer multiple comparisons test in Graphpad statistical software P < 0.05 = significant	Male Wistar rats [OUTB—Wistar, IND-cft [2c]] weighing 62 ± 5 g, Control: Rats fed one type of oil in their diet Experimental: Rats fed a blended oil in their diet.	Lipid analysis TC LDL-C HDL-C ALA EPA DHA Arachidonic Acid	Serum and liver lipids showed significant decrease in Total cholesterol [TC], Triglyceride [TG], LDL-C levels in GCO and GCO blended oil fed rats compared to native oil fed rats. ALA, EPA, DHA levels increased while linoleic acid [LA], arachidonic acid [AA] levels decreased in different tissues of GCO and GCO blended oils fed rats
12	[37] Vennila L, et al. 2012 Apr. To investigate the effect of sesamol on plasma and tissue lipid profiles in isoproterenol [ISO] - induced rats	1. Injecting isoproterenol [85mg/kg of body weight] subcutaneously for two days to induce myocardial infarction 2. Administer sesamol dissolved in 0.9% saline for seven days 3. Tissue collection on ninth day. Collected blood, plasma, heart, and liver. Statistical Analysis by: ANOVA Duncan’s Multiple Range Test [DMRT] using SPSS Software Package v.10.0. P ≤ 0.05 = significant	Adult male albino rats of the Wistar strain, weighing 180-200 g Control: Group I : Control [0.9% saline only] Group II : Control + sesamol [200 mg/kg BW] Group III : ISO control [85 mg/kg BW, sc, twice at an interval of 24 h on 1st and 2nd day] Experimental: Group IV : ISO + sesamol [50 mg/kg BW] Group V : ISO + sesamol [100 mg/kg BW] Group VI : ISO + sesamol [200 mg/kg BW]	Plasma lipid profile TC LDL VLDL HDL TG FFA PL Myocardial and Hepatic Tissue Lipids TC TG FFA PL Histopathological examination of liver tissues	Sesamol almost normalized serum ISO-administered effects [elevated of TC, LDL-C, VLDL-C and a reduced of HDL-C] and reduced plasma TG, FFA and PL significantly. ISO-administered rats showed significant increase in tissue TC, TG and FFA with a reduction in PL. Improvement was observed in the levels of these parameters to near normality in rats treated with sesamol. ISO-induced rat group showed dilated central vein surrounded by necrotic hepatocytes. Sesamol [50 mg/kg BW] treatment showed the central vein surrounded by normal hepatocytes with mild necrosis.
13	[38] Wu XL, et al. 2015 Aug. To test whether sesamol has anti-inflammatory activity in NF-kB and MAPK pathways in LPS-stimulated macrophages	1. RAW 264.7 macrophages were treated with sesamol, then LPS. 2. Pro-inflammatory cytokines were analyzed with ELISA 3. COX-2 and iNOS was analyzed by PCR 4. Nrf2 was analyzed by Western blots Statistical Analysis by: ANOVA Post hoc analyses with Dunnett’s test P < 0.05 = significant	RAW 264.7 macrophages from BCRC, Taiwan. Control: RAW 264.7 macrophages that were not treated with sesamol Experimental: RAW 264.7 macrophages that were treated with sesamol	Viability assay NO production ELISA to measure cytokines, chemokines, and PGE2 @ 450 nm Western Immunoblot to measure COX-2, HO-1, iNOS, Nrf2, IkB-a, Lamin B1, Phosphorylated-ikb-a, p65, adenosine monophosphate-activated protein kinase [AMPK], ERK1/2, p38, INK, phosphorylated-AMPK Real time PCR to evaluate gene expression Production of ROS	Sesamol inhibited production of nitric oxide, prostaglandin E2 [PGE2], and proinflammatory cytokines. Sesamol suppressed mRNA and protein expression of iNOS and COX-2, and enhanced antioxidant pathway represented by Nrf2 and HO-1. Sesamol suppressed NF-kB transport and decreased MAPK activation, but it increased AMPK activation.
14	[39] Sudhaker B, et al. 2011 Feb. To compare the effects of sesame oil and sunflower oil mix on patients taking Nifedipine.	1. Sesame and sunflower oil mix given to 4 groups of patients for 45 days. 2. Measured blood pressure lipid peroxidative markers, enzymatic and non-enzymatic antioxidants, lipid profiles and electrolytes in blood. Statistical Analysis by ANOVA Tukey’s multiple range tests on GraphPad Prism statical package ver. 5.0 P ≤ 0.05 = significant	14 normal male patients and 38 male patients [45-55 yrs old] with mild to moderate hypertension [SBP >140mmHG or DBP < 90mmHG] on treatment for nifedipine. Control: Normal patients Hypertensive patients on nifedipine Experimental: Hypertensive patients on nifedipine and sesame+sunflower oil mix.	Blood Pressure [mmHg] Lipid Profile TC [mg/dL] TGC [mg/dL] HDL [mg/dL] VLDL [mg/dL] LDL [mg/dL] TC/HDL Antioxidant status Superoxide dismutase [U*/mg Hb] Catalase [U*/mg Hb] Glutathione peroxidase [U*/mg Hb] Vitamin E [mg/dL] Vitamin C [mg/dL] Beta-carotene [mg/dL] Reduced glutathione [mg/dL] Lipid peroxidative markers Barbituric acid reactive substances [nmol/mL] Conjugated dienes [nmol/mL] Sodium [mEq/L] Potassium [mEq/L] Chloride [mEq/L]	Nifedipine and oil-mix patients showed a significant decrease in blood pressure, lipid peroxidative markers, lipid profile [excludes HDL levels], sodium, and chloride in comparison to nifedipine only patients. Nifedipine and oil-mix patients showed a significant increase in enzymatic antioxidants, non-enzymatic antioxidants, HDL, and potassium levels in comparison to nifedipine only patients.

Out of the 14 studies in Table 1, studies 4 and 14 were the only two studies using human subjects. Study 4 studied SO and its effects on endothelial function in hypertensive men. The results were that postprandial and long-term effects of SO increased flow-mediated dilation (FMD) from baseline comparison. The measured levels of intracellular adhesion molecules (ICAM) decreased significantly at 60 days in the long-term study but did not decrease significantly in the short-term postprandial study. The results of this study showed that SO has both local and short acting benefits such as vasodilation and also longer acting properties involved with the downregulation of the integrin ligand of the ICAM. Since the ICAM did not decrease significantly for the postprandial two-hour period, but did for the long-term period, this may suggest that the downregulation of the ICAM protein is due to the SO changing the gene expression of the ICAM gene. Study 14 was the second study out of the 14 studies that had human participants. The study was comparing nifedipine against nifedipine and an oil mix of SO and sunflower oil. Nifedipine and oil-mix patients showed a significant decrease in blood pressure, lipid peroxidative markers, lipid profile (excludes HDL levels), sodium, and chloride in comparison to nifedipine-only patients. Nifedipine and oil-mix patients showed a significant increase in enzymatic antioxidants, non-enzymatic antioxidants, HDL, and potassium levels in comparison to nifedipine-only patients.

The alteration of gene expression is supported by studies 1, 2, 3, 7, 9, and 13, which all show that SO’s mechanism of action includes the change in gene expression to inflammatory or lipid metabolism proteins. Study 1 found that mice livers showed increased expression of genes related to the reverse cholesterol transport (RCT) and lipid metabolism in SO-fed mice. Gene expression in mice aortas showed that SO-fed mice had increased mRNA levels of genes related to RCT but reduced levels of monocyte markers, ABCG, and scavenger receptors. Cytokines array showed that SO increased expression of genes pertaining to RCT and cholesterol metabolism. This indicates that SO is associated with increasing or decreasing of certain proteins. This study fed the mice the SO diet for 15 weeks, which is congruent with the idea of long-term gene expression changes. Study 1 is not the only study though that showed that SO could have short-term benefits also. Study 2 showed in mice not only that the expression of inflammatory mediators could be significantly reduced but also that the sesame oil aqueous extract (SOAE) could inhibit the oxidation of lipoproteins through lipopolysaccharides (LPS)-induced tumor necrosis factor (TNF)-α and interleukin (IL)-6. Study 3 examined SO and its ability to augment macrophage cholesterol efflux through a mitogen-activated protein kinase (MAPK) signaling pathway.

Study 5 examined α-lipoic acid and SO’s antioxidant protective property from diazinon (DZN) toxicity. The rats were sacrificed after four weeks, which showed that the combination of α-lipoic acid and SO was able to ameliorate the DZN intoxication. Study 6 showed that SO significantly decreased lipid peroxidation but did not significantly increase nitric oxide compared to n-acetyl cysteine. Also, the results found that 10% SO in the HFD for eight weeks in the mice did not decrease the lipid levels significantly compared to the control. The decrease in lipid peroxidation is consistent with the other studies that found that SO could affect lipid metabolism. Study 6 does not seem to be consistent with study 4, which showed both short- and long-term FMD. FMD is due to the sheer stress stimulus that produces a nitric oxide-dependent response. FMD is a direct marker of nitric oxide (NO) bioavailability and study 6 showed that only NAC restored NO bioavailability and not sesame oil supplementation. Study 4 was conducted on hypertensive males, and study 6 was conducted on mice. Study 7 showed that sesamol could decrease lipids and increase hepatic protein expression, while study 8 found that SO and not sesame seed could ameliorate high lipid levels and hepatic enzymes in rabbits fed a HFD. Studies 9 to 12 supported increased hepatic enzymes to decrease lipid levels. Study 13 was consistent with study 6 and found that sesamol inhibited production of NO and other proinflammatory markers.

## Review

In the studies that analyzed the effect of SO and its lignans on a lipid profile, SO has been shown to decrease TC, LDL, and VLDL plasma levels in hypercholesterolemic rodent and rabbit models. However, one reviewed study had stated that SO has no significant effects on lipid levels compared to the control [32]. Study 6 stated that SO treatment was not significantly different from the control treatment, which supported that the beneficial effects resulting from treatment with N-acetylcysteine (NAC) and SO together was only due to NAC; there was not enough SO to counteract the high cholesterol diet, which implies that SO treatment may be dose dependent. Other studies also agree that their research results that indicate the benefits of SO were truly significant at a certain dose [33-35]. Plant studies showed that plant stanols decreased LDL levels in a dose-dependent manner up to ~17% in a linear fashion when given up to 9 grams/milliliter [40]. The diet fed to the subjects in study 6 was composed of 10% SO compared to other studies that administered SO amounts depending on the weight of the subject. Depending on the weight of the subjects, 10% of SO in the diet may not have been enough to show SO’s anti-lipidemic effects [41]. Since caloric intake is higher than recommended in many populations, research has pointed out that consumption of supplemental oil should be associated with reduced intake of saturated fat [42]. This implies that SO could also be present in high amounts but not enough to induce its effects relative to the subject ingesting it.

In order to research additive effects of SO, some research explored the effects of mixed oils containing SO. Many vegetable oils, like sunflower or olive oils, also show hypocholesterolemic effects when ingested [43]. Research on the use of SO to treat atherosclerosis has yet to accumulate studies and research. So far, there are mixed reviews about SO, some studies stating that SO is not as effective as other edible oils. Study 6 studied the effects of SO in comparison to other oils in order to compare SO as a novel nutritional element to other vegetable oils that are part of our daily nutrition. Results in the study showed that SO was not as effective as the other oils [30]. This can be due to a few reasons, one being that the SO is not isolated and tested on its own. Blending different oils could result in a synergistic, antagonistic, or neutral effect. SO on its own can decrease cholesterol levels in the blood, but studies showed that SO blended with α-lipoic acids decreased cholesterol levels in the blood much more than α-lipoic acids or SO alone [31]. Another reason could be that there is no universal SO used throughout all the studies. There are many brands of SO, which are made with different techniques and compositions that may influence the effects of SO [44]. For example, SO is derived from heating sesame seeds to a certain temperature in order to create many of the lignans that are abundant in the oil but not the seeds [45]. This may increase or decrease the potency of SO compared to other vegetable oils and vice versa. In order to be able to properly compare the studies, one standardized concoction of SO should be created and consistently used throughout the studies.

Another way research explores the effects of SO is to test it in a mix with other components. For example, study 14 compared the effects of nifedipine to the effects of an oil mix of nifedipine with SO and sunflower oil on hypertensive patients, which resulted in a decrease in hypertensive factors. However, it is possible that the benefits could have been from the sunflower oil alone or that the SO had no benefit. It is also possible that the SO could have had adverse effects that negated some of the benefits of nifedipine or vice versa. The conclusion is that many of these studies are limited because they do not isolate the benefit of SO in humans alone and that different concentrations of SO are used in different studies and that a future study should examine the different concentrations of SO and its effects on humans with hyperlipidemia, hypertension, and diabetes mellitus. New studies can compare patients treated with differing mixes of medication, such as nifedipine, statins, metformin, with different concentrations of SO. Like statin therapy, many studies have promising results that show SO, when used as a dietary supplement, to lower LDL levels in the blood in coronary and aortic vasculature, as well as the liver and the brain [46-47]. The 14 studies did not examine the effects of SO on pancreatic beta cells, and future studies may want to see if SO has any potential effects since diabetes is also often associated with increased cardiovascular disease.

In most of the mentioned studies performed on animal models with SO, the benefits of SO and its components (e.g., sesaminol) can change the amount of gene transcription in different targets, such as endothelial cells, hepatocytes, and macrophages. Lignans such as sesamin and sesamol have gained popularity as they have been shown to have antioxidant and hypocholesterolemic effects [48-49]. However, many more studies need to be conducted on human participants since only two out of the 14 were on humans and one of the studies did not examine SO directly. In study 4, hypertensive men who took SO showed an increase in short- and long-term FMD and long-term ICAM. While these results look promising, there is a lot more that needs to be examined. In contrast, study 14 compared the benefits of SO and nifedipine with nifedipine alone. Neither of these studies compared the efficacy of SO to current pharmaceutical treatments in treating atherosclerosis. Previous studies have looked at the synergistic benefits as opposed to a direct comparison of SO with insulin-independent diabetic medications and anti-hypertensive medications [50]. These results show that in order to properly assess the effects of SO, there is a strong requirement for human trials with SO. Animal studies have shown that SO has decreased atherosclerotic factors without significant harm to the models, but it is unknown whether the same effects of SO would affect humans in the same way. In order to one day implicate SO and its effects on atherosclerotic patients, SO must past more clinical trials in order to gain traction as a possible treatment for atherosclerosis and other cardiovascular diseases.

## Conclusions

SO research shows promise in decreasing high levels of cholesterol and inflammation, lowering risks of atherosclerosis, and delaying the onset of cardiovascular diseases. Since SO is very inexpensive and natural, progressing research on SO to someday implement SO as a good pharmaceutical treatment would be an investment, especially when SO has yet to show adverse effects. However, SO has not had many clinical trials, and the benefits relative to other oils and medications still need to be investigated. This literature review found that the benefits of SO vary between studies due to the methodology of SO product, dose dependence, and examination of different variables. Many of these studies are limited because they do not isolate the benefit of SO in humans alone and because there are different concentrations of SO used in each study. Future studies should examine the different concentrations of SO and its effects on humans with hyperlipidemia, hypertension, and diabetes mellitus in a dose-dependent manner relative to the patient’s body habitus. Future studies can also look at synergism by comparing patients treated with differing combinations of medication, such as nifedipine, statins, metformin, with different concentrations of SO relative to the individual’s saturated fat diet.
